# Pubertal Development and Prepubertal Height and Weight Jointly Predict Young Adult Height and Body Mass Index in a Prospective Study in South Africa^1^,^2^

**DOI:** 10.3945/jn.116.231076

**Published:** 2016-06-22

**Authors:** Aryeh D Stein, Elizabeth A Lundeen, Reynaldo Martorell, Parminder S Suchdev, Neil K Mehta, Linda M Richter, Shane A Norris

**Affiliations:** 3 Rollins School of Public Health, Hubert Department of Global Health, and; 4 Nutrition and Health Sciences Program, Laney Graduate School, Emory University, Atlanta, GA; and; 5 Medical Research Council Developmental Pathways for Health Research Unit and; 6 Department of Science and Technology/National Research Foundation, Centre of Excellence of Human Development, University of the Witwatersrand, Johannesburg, South Africa

**Keywords:** adolescence, anthropometry, Birth to Twenty Plus study, human, latent class growth analysis, longitudinal study, puberty

## Abstract

**Background:** Height and adiposity track over childhood, but few studies, to our knowledge, have longitudinally examined the mediating relation of the timing and progression of puberty.

**Objective:** We assessed interrelations between prepubertal height and body mass index, the progression through puberty, and young adult height and adiposity.

**Methods:** We analyzed data from the Birth to Twenty Plus study (females, *n* = 823; males, *n* = 765). Serial measures of anthropometry and pubertal development were obtained between ages 9 and 16 y. We used latent class growth analysis to categorize pubertal development with respect to pubic hair (females and males), breasts (females), and genitalia (males) development. Adult height and weight were obtained at ages 18 to 20 y.

**Results:** Among females, higher latent class (earlier initiation and faster progression through puberty) was associated with an increased risk of obesity [pubic hair class 3 compared with class 1: RR, 3.41 (95% CI: 1.57, 7.44)] and inconsistent associations with height. Among males, higher latent class was associated with increased adult height [pubic hair development class 3 compared with class 1: 2.43 cm (95% CI: 0.88, 4.00)] and increased risk of overweight/obesity [pubic hair development class 3 compared with class 1: OR, 3.44 (95% CI: 1.44, 8.20)]. In females, the association with adult height became inverse after adjusting for prepubertal height [pubic hair development class 3 compared with class 1: females, −1.31 cm (95% CI: −2.32, −0.31)]; in males, the association with height was attenuated with this adjustment [−0.56 cm (95% CI: −1.63, 0.52)]. Associations with adiposity were attenuated after adjusting for prepubertal adiposity.

**Conclusions:** Progression through puberty modifies the relation between prepubertal and adult anthropometry. Screening for early or rapid progression of puberty might identify children at an increased risk of becoming overweight or obese adults.

## Introduction

Puberty is a period of rapid change in stature and body composition, with both males and females experiencing acceleration in linear growth velocity (1). Puberty leads to sexual dimorphism, with males gaining greater amounts of muscle and skeletal mass and females acquiring more fat mass (1). The age of pubertal onset has been associated with adult anthropometric outcomes—girls who experience menarche at younger ages have reduced adult height, greater BMI, and an increased risk of obesity during adulthood (2–8).

It is not known whether the age of pubertal onset is causally related to adult anthropometric outcomes. The association between the age of pubertal onset and adult BMI may be explained by higher adiposity during childhood and early adolescence. Numerous studies have shown that a higher BMI during childhood or prepubescent adolescence is associated with an earlier age of pubertal onset in girls (9–18). Other studies have shown that childhood BMI tends to track into adulthood (7, 13, 19–20). However, to our knowledge, few studies that link early puberty to higher adult BMI have been able to address the longitudinal nature of this relation (7). Whether the relation between age at menarche and adult BMI can be explained by childhood BMI remains unclear (7, 8, 22). Prepubertal height is positively correlated with adult height (24). The relation between age of pubertal onset and adult height may be explained by this correlation. Delaying puberty or slowing its progression might result in greater attained adult height by allowing additional time for growth to occur.

Given secular trends toward earlier menarcheal age (25, 26) and earlier pubertal development among girls (16, 27–30) and boys (29–31), it is important to better understand the mechanisms that relate pubertal development to adult anthropometric outcomes. The secular trends have also occurred concurrently with a global rise in overweight and obesity in adults (32), giving rise to speculation that they could be linked (7).

The relation between pubertal development and adult anthropometric outcomes has been primarily studied among girls and has focused on age at menarche. This relation has been less studied among boys, and much less is known about this relation in the context of other indicators of puberty, such as the development of secondary sexual characteristics like breasts, genitals, and pubic hair. Furthermore, to our knowledge, few studies have explored the process of pubertal development in relation to adult anthropometric outcomes or have had the longitudinal data necessary to account for the role of prepubertal anthropometric measures.

We address these gaps with the use of longitudinal data from a South African cohort of females and males who were followed prospectively through puberty to young adulthood to explore the relation of young adult height and BMI with several indicators of pubertal development, including age at menarche and the timing and tempo of the development of secondary sexual characteristics.

## Methods

### 

#### Setting.

We analyzed data from the Birth to Twenty Plus study, a birth cohort initiated in 1990 in Soweto and Johannesburg, South Africa. Detailed information on this cohort has been published elsewhere (33). The cohort enrolled 3273 children who were born during a 6-wk period in early 1990. Participants were predominantly black and from families with a low socioeconomic status. The prospective study was designed to track the growth, health, well being, and educational achievement of the children. Throughout the study, participants or their caregivers provided written informed consent, and ethical approval was obtained from the University of the Witwatersrand Committee for Research on Human Subjects. At birth and in the months immediately following birth, data on demographic and socioeconomic variables, such as the child’s ethnicity and household assets, were collected. We categorized ethnicity as black compared with other and categorized household wealth into quintiles, with an additional category for missing information.

#### Anthropometry.

At each study visit, weight and height were measured with the use standard procedures (34). BMI was calculated as kg/m^2^, and childhood measures were converted to *z* scores with the use of the age-appropriate WHO reference (35, 36). To maximize the available sample size and reduce the probability that the child would already be peripubertal at the time of measurement, we characterized prepubertal height and BMI *z* scores in decreasing order of preference with the use of measures obtained at age 5, 8, or 9 y (to avoid obtaining both prepubertal measures of anthropometry and the base for the pubertal trajectory at the same age). At adulthood (considered herein to be the latest measure obtained at the study visits between ages 18 and 20 y), participants with a BMI <25.0 were classified as normal or underweight, those with a BMI 25.0–29.9 as overweight but not obese, and those with a BMI ≥30.0 as obese (37–39).

#### Pubertal progression.

At ages 9–16 y, pubertal development was assessed with the Tanner sexual maturation scale (SMS) (40). The scale consisted of 5 progressive stages in the development of secondary sexual characteristics—breasts in females, genitals in males, and pubic hair in both sexes (41, 42). The stages ranged from 1 (prepubertal) to 5 (postpubertal). The drawings were accompanied by written descriptions of each stage. From ages 9–11 y, a trained health care professional administered the Tanner SMS. From ages 12–16 y, participants self-rated their pubertal development after self-assessment was validated against expert ratings in a comparable group of South African adolescents (43). Girls self-reported the onset of menses at each survey wave.

We characterized pubertal progression in 2 ways. First, as described elsewhere, we performed latent class growth analysis to group participants into distinct classes based on a common pubertal developmental trajectory, which can be characterized based on the age at which pubertal progression started and the rate at which it progressed (18). We used all available data for ages 9–16 y. We conducted separate analyses for pubic hair (females and males), breasts (females), and genitalia (males). We identified 3 trajectory classes for pubic hair development for both females and males and 4 classes for breast (females) and genital (males) development. The classes were numbered 1–3 for pubic hair and 1–4 for breast and male genitalia, with 1 representing the class with the slowest progression through puberty.

We also categorized pubertal status at age 12 y, an age at which we expected substantial variability in the Tanner SMS stage. Children who were at the modal score of pubic hair, breast, and genital development were categorized as being at a medium level. Those with a lower score were considered later maturers, and those with a higher score were considered earlier maturers. Because not all children were assessed at age 12 y, we also developed a category of missing (28.7–31.1% of observations across the measures) and included this stratum in the analysis.

#### Inclusion in the analysis.

Participants were included in this analysis if they had ≥1 Tanner SMS measurement between 9 and 16 y, as well as valid height and weight measurements at young adulthood (18–20 y). This resulted in an analytical sample of 1918 participants (990 females and 928 males; 58.6% of the original enrolled sample), of whom 823 females and 765 males also had ≥1 measure of prepubertal height and BMI.

#### Statistical analysis.

Analyses were stratified by sex. We tested whether adult mean height and BMI and the distribution of overweight/obese categories differed across pubertal development trajectory classes and across categories of pubertal status at 12 y with the use of ANOVA and chi-square as appropriate.

We used linear regression to estimate the relation of pubertal development trajectory classes or pubertal status at age 12 y (females and males) and of age at menarche (females only) with adult height. We used logistic regression to estimate the relation of pubertal development trajectory classes or pubertal status at age 12 y (females and males) and of age at menarche (females only) with adult adiposity. For females, we used multinomial logistic regression and characterized adult adiposity as normal, overweight, or obese. For males, we used binomial logistic regression and a combined overweight/obesity category because there were not enough males who were overweight or obese to permit further categorization. RRs are presented for the multinomial logistic regression models, and ORs are presented for the logistic regression models.

We developed a series of models and adjusted incrementally for ethnicity and wealth (model 1) and prepubertal height and BMI *z* scores (model 2). To examine whether the presence or absence of prepubertal data might introduce bias, we repeated model 1 for the whole sample and for those for whom prepubertal data were available; because there were trivial differences between the estimates, only the latter are presented in the tables.

We used Mplus version 7.3 (Muthén & Muthén) to perform the latent class growth analysis and Stata version 14.0 (StataCorp) for all other analyses. *P* < 0.05 was considered to be statistically significant. 

## Results

The study sample was 52% female (**Table 1**). Most participants (85%) were black. Compared to international references, the children were short before pubertal onset (mean HR: females, −0.66; males, −0.69), with mean BMI *z* scores very close to the WHO reference. At age 12 y, 30.0% and 26.2% of females were in Tanner stage 1 or 2 for pubic hair and breasts, respectively, whereas 16.8% and 15.7% of males were in Tanner stage 1 for pubic hair and genitalia, respectively. The mean age at menarche was 12.7 y. The mean adult height was 159.6 cm among females and 171.2 cm among males, and mean BMI was 23.1 among females and 20.6 among males. In all, 19% of females were overweight and 8.7% were obese. Among males, 5.9% were overweight and 2.6% were obese. There were trivial differences across a range of variables between the whole sample and for those for whom prepubertal data were available (Table 1).

**TABLE 1:** Selected characteristics of the Birth to Twenty Plus study cohort^1^

	Females		Males	
	All (*n* = 990)	Subset with prepubertal data (*n* = 823)	All (*n* = 928)	Subset with prepubertal data (*n* = 765)
Ethnicity				
Black	84.7	84.1	85.0	83.4
Other	15.3	15.9	15.0	16.6
Household wealth				
Category 1	13.3	12.3	15.1	13.1
Category 2	15.6	16.2	15.4	15.6
Category 3	30.8	31.0	30.1	30.3
Category 4	17.9	18.6	19.2	20.4
Category 5	13.2	14.1	11.9	12.6
Missing	9.2	7.9	8.4	8.1
Prepubertal height,^2^ *z* score		−0.66 ± 0.97		−0.69 ± 1.00
Prepubertal BMI,^2^ *z* score		−0.03 ± 0.96		0.00 ± 1.03
Puberty latent growth class^3^				
Pubic hair				
Class 1	32.8	32.8	28.2	27.8
Class 2	54.7	54.8	58.4	58.2
Class 3	12.5	12.4	13.4	14.0
Breasts/genitals				
Class 1	23.3	22.5	5.7	6.0
Class 2	25.5	25.9	36.3	36.1
Class 3	36.7	37.9	50.2	49.5
Class 4	14.6	13.7	7.8	8.4
Pubertal stage at 12 y^4^				
Pubic hair				
Earlier	13.8	14.0	21.7	22.7
Medium	27.1	27.1	31.0	33.7
Later	30.0	30.5	16.8	16.5
Missing	29.1	28.4	30.5	27.1
Breasts/genitals				
Earlier	16.5	16.2	22.4	23.1
Medium	28.7	29.0	31.1	33.3
Later	26.2	26.9	15.7	16.2
Missing	28.7	28.0	30.7	27.3
Adult height, cm	160 ± 6.2	160 ± 6.2	171 ± 6.7	171 ± 6.7
Adult BMI, kg/m^2^	23.1 ± 4.7	23.1 ± 4.6	20.6 ± 3.5	20.8 ± 3.7
Adult BMI status				
Normal or underweight	72.2	72.5	91.5	90.6
Overweight	19.1	19.1	5.9	6.3
Obese	8.7	8.4	2.6	3.1
Age at menarche, y	12.7 ± 1.2	12.7 ± 1.2	—	—

1 Values are means ± SDs or percentages unless otherwise noted.

2 Based on data at ages 5, 8, or 9 y; *n* = 245, 545, and 33 and 204, 540, and 21 for females and males, respectively.

3 Classes derived from latent class growth models with the use of data from participants aged 9–16 y. Classes were ordered such that class 1 represents individuals who started puberty later and/or progressed more slowly.

4 Tanner stage at 12 y relative to peers—pubic hair for females: earlier maturation (stage 4 or 5), medium maturation (stage 3), and later maturation (stage 1 or 2); pubic hair for males: earlier maturation (stage 3, 4, or 5), medium maturation (stage 2), and later maturation (stage 1); breast development for females: earlier maturation (stage 4 or 5), medium maturation (stage 3), and later maturation (stage 1 or 2); and genital development for males: earlier maturation (stage 3, 4, or 5), medium maturation (stage 2), and later maturation (stage 1).

In unadjusted analyses among females, adult height was not significantly associated with trajectory class membership for pubic hair development; for breast development there was an overall significant association with no clear pattern (**Table 2**). For both pubic hair and breast development trajectory class, increasing class membership (i.e., faster progression through puberty) was associated with higher adult BMI (*P* < 0.001). The prevalence of obesity increased from 5.2% to 17.7% (*P* < 0.001) across the classes of pubic hair development and from 4.3% to 20.8% (*P* < 0.001) across classes of breast development.

**TABLE 2:** Mean height and BMI and percentage of overweight and obese among young adults according to pubertal development trajectory class or Tanner stage at 12 y^1^

	Women (*n* = 990)				Men (*n* = 928)		
	Height, cm	BMI, kg/m^2^	Overweight^2^	Obese^3^	Height, cm	BMI, kg/m^2^	Overweight/obese^4^
Trajectory classes^5^						
Pubic hair							
Class 1	159 ± 6.1	22.5 ± 4.6	19.1	5.2	170 ± 6.3	20.0 ± 3.2	3.8
Class 2	160 ± 6.3	23.1 ± 4.7	18.5	8.7	172 ± 6.5	20.6 ± 3.6	8.7
Class 3	160 ± 6.3	24.7 ± 4.9	21.8	17.7	173 ± 7.6	22.1 ± 3.5	17.7
*P* value^6^	0.88	<0.001	0.001	0.001	<0.001	<0.001	<0.001
Breasts/genitals							
Class 1	160 ± 6.1	21.7 ± 4.4	11.3	4.3	170 ± 6.1	19.9 ± 3.6	7.6
Class 2	160 ± 6.6	22.3 ± 3.6	18.2	3.6	170 ± 6.5	20.5 ± 3.4	6.8
Class 3	159 ± 6.0	23.5 ± 5.1	19.8	10.2	172 ± 6.5	20.7 ± 3.7	9.4
Class 4	160 ± 6.1	25.8 ± 4.7	31.3	20.8	173 ± 7.8	21.5 ± 2.6	11.1
*P* value	0.036	<0.001	<0.001	<0.001	<0.001	0.051	0.49
Pubertal development stage at 12 y^7^						
Pubic hair							
Earlier	160 ± 6.4	24.4 ± 5.1	18.3	16.1	172 ± 7.0	21.4 ± 4.0	13.4
Medium	160 ± 6.0	23.2 ± 4.2	23.1	7.5	171 ± 6.3	20.5 ± 3.4	6.2
Later	160 ± 6.2	23.0 ± 5.0	18.9	7.7	170 ± 6.6	19.9 ± 2.8	5.1
Missing	160 ± 6.5	22.5 ± 4.5	16.0	7.3	172 ± 6.9	20.6 ± 3.6	9.2
*P* value	0.85	0.002	0.015	0.015	0.18	<0.001	0.014
Breasts/genitals							
Earlier	159 ± 6.3	24.3 ± 5.0	22.7	14.1	171 ± 6.9	21.2 ± 4.2	10.6
Medium	160 ± 6.1	23.8 ± 5.0	22.9	10.2	171 ± 6.3	20.4 ± 3.0	6.6
Later	160 ± 6.1	22.2 ± 4.1	15.8	5.0	170 ± 6.7	20.4 ± 3.3	8.2
Missing	159 ± 6.5	22.5 ± 4.6	16.1	7.4	172 ± 6.8	20.6 ± 3.6	9.1
*P* value	0.078	<0.001	<0.001	<0.001	0.17	0.096	0.44

1 Values are means ± SDs or percentages unless otherwise noted. There was an insufficient number of males who were overweight or obese to model the risk of these outcomes separately.

2 BMI (in kg/m^2^): 25.0–29.9.

3 BMI (in kg/m^2^): ≥30.0.

4 BMI (in kg/m^2^): ≥25.0.

5 Classes derived from latent class growth models. Classes were ordered such that class 1 represents individuals who started puberty later and/or progressed more slowly, and increasing class numbers represents earlier initiation and faster tempo of puberty.

6 ANOVA used for adult height and BMI; chi-square used for overweight/obesity categories.

7 Tanner stage at 12 y relative to peers—pubic hair for females: earlier maturation (stage 4 or 5), medium maturation (stage 3), and later maturation (stage 1 or 2); pubic hair for males: earlier maturation (stage 3, 4, or 5), medium maturation (stage 2), and later maturation (stage 1); breast development for females: earlier maturation (stage 4 or 5), medium maturation (stage 3), and later maturation (stage 1 or 2); genital development for males: earlier maturation (stage 3, 4, or 5), medium maturation (stage 2), and later maturation (stage 1).

Among males, mean adult height increased across pubic hair and genital trajectory classes (both *P* < 0.001) (Table 2). Mean adult BMI also increased across classes of pubic hair (*P* < 0.001) and genital (*P* = 0.051) development trajectory classes. The prevalence of overweight/obesity increased from 3.8% to 17.7% (*P* < 0.001) across the classes of pubic hair development. No significant differences in the prevalence of overweight/obesity were observed across classes of genital development.

**Table 3** presents the regression results for adult height. The unadjusted results confirm the descriptive statistics presented in Table 2—no consistent association of pubertal development trajectory class membership in females. Conversely, in males adult height was higher in individuals in the higher pubic hair or genital development trajectory classes. Adjusting for ethnicity and household wealth (with or without restriction to the sample for whom prepubertal height and BMI were available) attenuated the estimates. Adjusting additionally for prepubertal height and BMI (model 2) resulted in considerable changes in the estimates, suggesting that once prepubertal size is known, faster progression through puberty can be observed with lower attained height. The estimates were significant (*P* < 0.05) for females and suggestive in males. The pattern for pubertal status at age 12 y was less consistent. Each year of later onset of menarche was associated with 0.58 cm (in the unadjusted model, *P* < 0.001) to 1.15 cm (in the fully adjusted model, *P* < 0.001) greater adult height.

**TABLE 3:** Differences in adult height across pubertal development trajectory class or Tanner stage at 12 y^1^

	Women (*n* = 990)			Men (*n* = 928)		
	Unadjusted	Model 1^2^	Model 2^3^	Unadjusted	Model 1	Model 2
Trajectory classes^4^						
Pubic hair						
Class 2	0.28 (−0.58, 1.14)	0.32 (−0.62, 1.27)	−0.63 (−1.29, 0.03)	1.99 (1.01, 2.96)	1.73 (0.63, 2.84)	0.45 (−0.29, 1.19)
Class 3	0.35 (−0.94, 1.65)	0.55 (−0.88, 1.98)	−1.31 (−2.32, −0.31)	3.15 (1.73, 4.56)	2.43 (0.86, 4.00)	−0.56 (−1.63, 0.52)
Breasts/genitals						
Class 2	−0.14 (−1.25, 0.97)	0.15 (−1.08, 1.38)	0.12 (−0.71, 0.96)	−0.40 (−2.32, 1.51)	0.08 (−2.02, 2.18)	−0.58 (−1.98, 0.83)
Class 3	−1.34 (−2.36, −0.31)	−0.76 (−1.90, 0.38)	−1.83 (−2.62, −1.05)	1.75 (−0.13, 3.63)	1.95 (−0.11, 4.01)	−0.38 (−1.77, 1.01)
Class 4	−0.59 (−1.89, 0.70)	0.13 (−1.33, 1.60)	−2.07 (−3.10, −1.05)	2.32 (−0.02, 4.67)	2.11 (−0.45, 4.66)	−1.51 (−3.24, 0.23)
Pubertal stage at 12 y^5^						
Pubic hair						
Earlier	−0.08 (−1.36, 1.21)	0.79 (−0.62, 2.19)	−0.75 (−1.72, 0.23)	0.71 (−0.50, 1.91)	0.53 (−0.77, 1.84)	−0.78 (−1.65, 0.09)
Later	0.04 (−0.99, 1.07)	0.15 (−0.98, 1.27)	1.45 (0.67, 2.22)	−0.68 (−1.98, 0.62)	−0.66 (−2.10, 0.78)	0.13 (−0.83, 1.09)
Missing	−0.37 (−1.42, 0.67)	−0.44 (−1.63, 0.75)	0.48 (−0.33, 1.30)	0.56 (−0.54, 1.66)	0.02 (−1.30, 1.35)	0.13 (−0.75, 1.01)
Breasts/genitals						
Earlier	−1.32 (−2.52, −0.12)	−0.82 (−2.14, 0.50)	−1.50 (−2.41, −0.60)	0.17 (−1.03, 1.36)	−0.03 (−1.34, 1.27)	−0.77 (−1.63, 0.10)
Later	−0.01 (−1.06, 1.04)	0.17 (−0.97, 1.31)	1.54 (0.76, 2.33)	−1.06 (−2.39, 0.27)	−1.11 (−2.56, 0.34)	0.02 (−0.95, 0.98)
Missing	−0.79 (−1.82, 0.24)	−0.92 (−2.09, 0.25)	0.06 (−0.74, 0.86)	0.43 (−0.67, 1.52)	−0.07 (−1.39, 1.24)	0.22 (−0.65, 1.09)
Age at menarche, y	0.58 (0.26, 0.90)	0.43 (0.08, 0.78)	1.15 (0.91, 1.39)	—	—	—

1 All values are cm (95% CIs).

2 Adjusted for ethnicity (black compared with other) and quintiles of household wealth; *n* = 823 (women) and 765 (men).

3 Additionally adjusted for prepubertal height and BMI; *n* = 823 (women) and 765 (men).

4 There were 3 growth trajectory classes for pubic hair development (females and males) and 4 growth trajectory classes for breast development (women) and genital development (men). The reference category is class 1 (slowest progression).

5 Tanner stage at 12 y relative to peers—pubic hair and breast development (females): earlier maturation (stage 4 or 5), medium maturation (stage 3), later maturation (stage 1 or 2); pubic hair and genital development (males): earlier maturation (stage 3, 4, or 5), medium maturation (stage 2), later maturation (stage 1). Reference category is medium maturation.

**Table 4** presents the regression results for adult overweight and obesity. In females, increasing pubertal development trajectory class membership was associated with an increased risk of overweight, especially of obesity. In males in whom overweight/obesity were combined, increasing pubertal development trajectory class membership was associated with an increased risk of the combined category. Adjusting for ethnicity and household wealth attenuated the estimates slightly, whereas adjusting for prepubertal height and BMI further attenuated the estimates—generally down to null. Pubertal status at 12 y (coded as early, at, or later than the sample modal value) was not consistently associated with overweight or obesity. In females, age at menarche was inversely associated with adult overweight and obesity; the estimates were attenuated after adjusting for prepubertal height and BMI.

**TABLE 4:** Adult overweight and obesity according to pubertal development trajectory class or Tanner stage at 12 y^1^

	Women (*n* = 990)								
	BMI 25.0–29.9^2^			BMI ≥ 30.0			Men (*n* = 928)^3^		
	Unadjusted	Model 1^4^	Model 2^5^	Unadjusted	Model 1	Model 2	Unadjusted	Model 1	Model 2
Trajectory classes^6^									
Pubic hair									
Class 2	1.01 (0.71, 1.44)	1.02 (0.69, 1.51)	0.92 (0.60, 1.39)	1.73 (0.97, 3.07)	1.62 (0.87, 3.03)	1.28 (0.61, 2.66)	2.39 (1.19, 4.81)	2.21 (1.05, 4.67)	1.84 (0.81, 4.21)
Class 3	1.43 (0.85, 2.40)	1.46 (0.82, 2.59)	0.98 (0.53, 1.81)	4.24 (2.14, 8.41)	3.41 (1.57, 7.44)	1.75 (0.71, 4.32)	5.44 (2.49, 11.9)	3.44 (1.44, 8.20)	2.00 (0.76, 5.28)
Breasts/genitals									
Class 2	1.75 (1.04, 2.95)	1.72 (0.97, 3.07)	1.77 (0.97, 3.21)	0.89 (0.35, 2.24)	0.73 (0.26, 2.01)	1.01 (0.33, 3.10)	0.90 (0.30, 2.71)	1.10 (0.31, 3.92)	0.59 (0.15, 2.27)
Class 3	2.13 (1.31, 3.46)	2.00 (1.17, 3.43)	1.85 (0.94, 2.91)	2.84 (1.38, 5.85)	2.40 (1.11, 5.20)	1.50 (0.61, 3.69)	1.28 (0.44, 3.71)	1.28 (0.37, 4.41)	1.05 (0.29, 3.83)
Class 4	4.89 (2.81, 8.52)	4.68 (2.50, 8.73)	3.63 (1.87, 7.03)	8.48 (3.94, 18.3)	7.04 (3.03, 16.4)	4.60 (1.71, 12.3)	1.53 (0.44, 5.38)	1.21 (0.28, 5.22)	0.64 (0.14, 2.94)
Pubertal stage at 12y^7^									
Pubic hair									
Earlier	0.83 (0.49, 1.41)	0.75 (0.42, 1.35)	0.57 (0.31, 1.06)	2.27 (1.18, 4.38)	1.84 (0.89, 3.78)	1.24 (0.53, 2.93)	2.33 (1.24, 4.35)	2.04 (1.05, 3.97)	1.86 (0.87, 3.97)
Later	0.77 (0.51, 1.16)	0.75 (0.48, 1.17)	0.82 (0.51, 1.33)	0.98 (0.52, 1.84)	0.81 (0.41, 1.62)	0.86 (0.37, 1.99)	0.81 (0.34, 1.91)	0.89 (0.36, 2.23)	1.07 (0.39, 2.99)
Missing	0.62 (0.41, 0.96)	0.68 (0.40, 1.08)	0.55 (0.33, 0.93)	0.88 (0.46, 1.68)	0.92 (0.44, 1.90)	0.68 (0.29, 1.60)	1.52 (0.81, 2.83)	1.36 (0.66, 2.79)	1.61 (0.69, 3.74)
Breasts/genitals									
Earlier	1.05 (0.66, 1.68)	1.23 (0.64, 2.35)	0.86 (0.50, 1.50)	1.46 (0.80, 2.66)	1.10 (0.30, 4.10)	0.89 (0.39, 2.00)	1.68 (0.88, 3.19)	1.34 (0.68, 2.65)	1.37 (0.63, 2.99)
Later	0.58 (0.38, 0.91)	0.74 (0.41, 1.35)	0.69 (0.41, 1.14)	0.42 (0.21, 0.82)	0.92 (0.25, 3.38)	0.48 (0.20, 1.15)	1.27 (0.60, 2.70)	1.18 (0.52, 2.65)	1.77 (0.72, 4.36)
Missing	0.62 (0.41, 0.95)	0.44 (0.22, 0.89)	0.58 (0.35, 0.98)	0.63 (0.35, 1.15)	1.76 (0.52, 5.93)	0.55 (0.25, 1.22)	1.43 (0.77, 2.64)	1.16 (0.58, 2.33)	1.45 (0.64, 3.28)
Age at menarche, y	0.83 (0.73, 0.95)	0.80 (0.69, 0.93)	0.94 (0.79, 1.12)	0.62 (0.52, 0.76)	0.66 (0.53, 0.82)	0.83 (0.63, 1.10)	—	—	—

1 Values are RRs (95% CIs) for women and ORs (95% CIs) for men, respectively.

2 Multinomial logistic regression comparing the RRs of overweight but not obese [BMI (in kg/m^2^): 25.0–29.9] and obese (BMI : ≥30.0) to normal or underweight adults (BMI: <25.0).

3 Logistic regression comparing the ORs of overweight or obese [BMI (in kg/m^2^) ≥25.0] to normal or underweight adults (BMI: <25.0).

4 Adjusted for ethnicity (black compared with other) and quintiles of household wealth; *n* = 823 (women) and 765 (men).

5 Additionally adjusted for prepubertal height and BMI; *n* = 823 (women) and 765 (men).

6 There were 3 growth trajectory classes for pubic hair development (women and men) and 4 growth trajectory classes for breast development (women) and genital development (men). The reference category is class 1 (slowest progression).

7 Tanner stage at 12 y relative to peers—pubic hair and breast development (women): earlier maturation (stage 4 or 5), medium maturation (stage 3), and later maturation (stage 1 or 2); and pubic hair and genital development (men): earlier maturation (stage 3, 4, or 5), medium maturation (stage 2), and later maturation (stage 1). The reference category is medium maturation.

## Discussion

In this prospectively followed contemporary South African cohort, we explored the relation between several indicators of pubertal development and young adult height and adiposity as measured by BMI. In males, but not in females, membership in a higher pubertal development trajectory class was associated with increased adult height, an association that was reversed when controlling for prepubertal height and BMI. For both males and females, membership in a higher pubertal development class was associated with increased BMI and prevalence of overweight and obesity in young adulthood. These associations were attenuated after adjusting for prepubertal height and BMI. Age at menarche was positively associated with attained height and inversely associated with a risk of overweight and obesity in young adulthood.

Given secular trends showing a reduction in menarcheal age (25, 26) and earlier development of breasts and pubic hair among girls (16, 27–30) and earlier genital and pubic hair development in boys (29–31), it is critical to better understand the nature of the relation between prepubertal size, pubertal development, and adult anthropometric outcomes. Our data contribute to the literature concerning the growth and development of contemporary cohorts and specifically suggest that rapid passage through puberty is associated with a lower final attained height and higher BMI. Unique features of our study include multiple waves of data collected before, during, and after puberty; the relatively high retention of the cohort from birth through young adulthood; and the inclusion of both men and women.

The literature on pubertal development and adult anthropometric outcomes has primarily focused on females [Sandhu et al. (44) is an exception], with the measure of puberty generally defined as age at menarche. Several studies have shown that early menarche is associated with shorter adult stature (2–4, 45). As the pace of linear growth declines considerably after puberty, girls who experience earlier menarche may miss out on several years of rapid prepubertal growth, resulting in a reduced adult height (3). In addition, several studies have found that women who experience menarche at an earlier age have a greater adult BMI and substantially increased risk of obesity in adulthood (2–4, 6–8, 22, 45–47). The absence of a comparable measure of puberty in males has hindered research in this area, reinforcing the value of our data, with prospective ascertainment of Tanner scales from ages 9–16 y.

We have previously shown that height at ages 5 or 8 y and both BMI at 5 y and a rapid increase in BMI between 5 and 8 y predict pubertal trajectory class (18). In this study, we assessed to what extent our observed associations between pubertal class and adult height and adiposity could be explained by prepubertal height and adiposity. For adult height, we observed a strong impact of this adjustment, such that the sign on many of the estimates changed from positive to negative, especially for males. This would suggest that faster pubertal progression is in fact associated with lower final height once prepubertal height is taken into account. For BMI and for overweight and obesity, adjusting for prepubertal height and BMI attenuated but in general did not abolish the associations of pubertal development trajectory class with adiposity, suggesting that part of the association was attributable to the shared variance. The mechanisms behind these differing results for attained height and BMI are worthy of further investigation and may involve both genetic and behavioral aspects (48).

Measuring puberty can be challenging. The Tanner SMS ratings were self-reported at ages 12–16 y. The gold standard for pubertal staging is a physical examination by a trained clinician with the use of Tanner criteria (40). However, self-assessment with the use of the Tanner SMS has been validated in many populations, including a highly comparable population of South African adolescents (43) in which we have demonstrated that there was high concordance between adolescents’ and same-sex health professionals’ assessment of pubic hair growth and breast development in females (*k* coefficients 0.71 and 0.76, respectively) and pubic hair growth and genital development in males (*k* coefficients 0.63 and 0.60, respectively). From these results, we concluded that the Tanner pubertal self-rating seems to be a reasonably valid instrument to use among black South African youth provided the procedure is thoroughly explained to participants through the developed tutorial in the language of choice and privacy and confidentiality are assured. We followed this protocol in the longitudinal assessments of the Birth to Twenty Plus cohort. In addition, although measurement error associated with self-assessment could affect conclusions drawn about the level of pubertal development at any given cross-section within the study, the consistent use of self-rating from 12–16 y means that interpreting trends across time would be unlikely to be biased because measurement error is equally likely at all ages.

It has been suggested that the use of the Tanner SMS to characterize breast development in the absence of a physical examination by a clinician may result in measurement errors for overweight or obese girls (40). The Tanner SMS is most accurately used to describe female breast development when a trained clinician uses palpation to distinguish between actual breast tissue and adipose tissue. Overweight or obese females who use the Tanner self-assessment may be prone to overestimate their level of breast development because adipose tissue can be easily mistaken for breast tissue (40). However, the breast development rating does not simply rely on the size of the breast but also takes into account changes in the areola and nipple. Indeed, as part of the tutorial and the descriptions and pictures of the stages shown to female adolescents, these distinctions are explicitly highlighted.

BMI does not allow for differentiating between the relative contribution of fat mass and fat-free mass, and there may be differences in the extent to which BMI represents adiposity in males compared with females. In addition, overweight and obesity were relatively rare among the males in our sample (23), so some of the models had small sample sizes, resulting in imprecise estimates. Finally, our sample was primarily black and of low socioeconomic status.

Our results suggest that the relation of pubertal development to overweight and obesity is mostly accounted for by the influence of prepubertal BMI. Preventing childhood overweight and obesity is important in reducing adult obesity given strong tracking throughout life (24). Although the long-term consequences of variation in height caused by differences in pubertal progression are not known, it is known that adult obesity confers a greatly increased risk of chronic diseases such as diabetes and cardiovascular disease (49) and that understanding early risk factors for adult obesity is important in prevention efforts. Pubertal staging could be a useful screening tool in clinical practice because it provides an additional prediction of overweight and obesity in young adulthood.
